# STUDENTS’ LEARNING IN COMMUNITY-BASED PARTICIPATORY RESEARCH: THE POTTED PLANT PROJECT IN PROJECT SOAR

**DOI:** 10.1093/geroni/igz038.1505

**Published:** 2019-11-08

**Authors:** Deanna Dragan, Jermaine Mitchell, Rebecca S Allen, Pamela Payne-Foster, JoAnn Oliver, Christopher Spencer

**Affiliations:** 1 The University of Alabama, Tuscaloosa, Alabama, United States; 2 The University of Alabama School of Nursing, Tuscaloosa, Alabama, United States

## Abstract

Sharing Opinions and Advice about Research (Project SOAR), funded by PCORI, trained individuals living in under-resourced and underserved communities how to evaluate and provide advice to scientists about recruitment procedures, survey items, and intervention components for implementation in their communities. Moreover, graduate students learned community-based participatory research (CBPR) procedures and interacted with communities in implementing their own research projects. Students worked with the urban Holt community in western Alabama on issues of food insecurity due to pollution and concerns about growing vegetables and herbs in the soil. Students participated in the Potted Plant Project and plant give away, collecting questionnaire and health data. Students reported finding the fluid nature of research during this community event both stressful and rewarding. Students also identified how familiarity with CBPR procedures enhanced their clinical service provision in the community. Discussion will focus on future graduate training needs in implementation of CBPR.

